# Anxiety, depression and HIV in older gay and bisexual men

**DOI:** 10.1192/j.eurpsy.2021.505

**Published:** 2021-08-13

**Authors:** H. Pereira, I. Batista

**Affiliations:** 1 Ubi, Research Centre in Sports Sciences, Health Sciences and Human Development, Covilha, Portugal; 2 Cics-ubi, Centre for Research in Health Sciences, Covilha, Portugal; 3 Psychology And Education, University of Beira Interior, Covilha, Portugal

**Keywords:** HIV, Aging, Anxiety, Depression

## Abstract

**Introduction:**

Negative consequences of social homonegativity and HIV status among older gay and bisexual men in Portugal are still to be documented.

**Objectives:**

This study seeks to evaluate depression and anxiety disparities among older gay and bisexual men, based upon their HIV status.

**Methods:**

This is a quantitative, descriptive, comparative, and cross-sectional study. Responses were collected from a total of 201 men, with 16.9% being HIV positive, 80.6% identifying as homosexual and 13.9% identifying as bisexual. Participants responded to the Rosenberg Self-Esteem Scale, the BSI-18, and the CDRISC-10.

**Results:**

The sample collected revealed moderate levels of depression and anxiety that were below the average observed among the general population. Older gay men showed higher levels of depression when compared to older bisexual men. Self-esteem was negatively correlated with both depression and anxiety while being positively correlated with resilience. In contrast, depression was negatively correlated with resilience and, conversely, positively correlated with anxiety. Furthermore, anxiety was negatively correlated with resilience. Multiple linear regression models explain 33% of the general variation of depressive symptoms and 25% of the variation of symptoms of anxiety. Regarding comparisons based on HIV status, no statistically significant differences were found between HIV-positive and HIV-negative men.

**Conclusions:**

This study offers a pioneering contribution to the literature on aging and mental health among older sexual minorities in Portugal, giving a voice to older HIV-positive and HIV-negative GBM in Portugal, in order to gain a better understanding of the barriers and obstacles that they face throughout the aging process.

